# Comparison of analyses of the XV^th ^QTLMAS common dataset III: Genomic Estimations of Breeding Values

**DOI:** 10.1186/1753-6561-6-S2-S3

**Published:** 2012-05-21

**Authors:** Pascale Le Roy, Olivier Filangi, Olivier Demeure, Jean-Michel Elsen

**Affiliations:** 1 INRA, UMR1348 PEGASE, Domaine de la Prise, 35590 Saint-Gilles, France; 2 Agrocampus OUEST, UMR1348 PEGASE, 65 rue de St Brieuc, 35042 Rennes, France; 3 INRA UR0631 SAGA, chemin de borde rouge, BP 52627, 31326 Castanet-Tolosan, France

## Abstract

**Background:**

The QTLMAS XV^th ^dataset consisted of pedigree, marker genotypes and quantitative trait performances of animals with a sib family structure. Pedigree and genotypes concerned 3,000 progenies among those 2,000 were phenotyped. The trait was regulated by 8 QTLs which displayed additive, imprinting or epistatic effects. The 1,000 unphenotyped progenies were considered as candidates to selection and their Genomic Estimated Breeding Values (GEBV) were evaluated by participants of the XV^th ^QTLMAS workshop. This paper aims at comparing the GEBV estimation results obtained by seven participants to the workshop.

**Methods:**

From the known QTL genotypes of each candidate, two "true" genomic values (TV) were estimated by organizers: the genotypic value of the candidate (TGV) and the expectation of its progeny genotypic values (TBV). GEBV were computed by the participants following different statistical methods: random linear models (including BLUP and Ridge Regression), selection variable techniques (LASSO, Elastic Net) and Bayesian methods. Accuracy was evaluated by the correlation between TV (TGV or TBV) and GEBV presented by participants. Rank correlation of the best 10% of individuals and error in predictions were also evaluated. Bias was tested by regression of TV on GEBV.

**Results:**

Large differences between methods were found for all criteria and type of genetic values (TGV, TBV). In general, the criteria ranked consistently methods belonging to the same family.

**Conclusions:**

Bayesian methods - A<B<C<Cπ - were the most efficient whatever the criteria and the True Value considered (with the notable exception of the MSEP of the TBV). The selection variable procedures (LASSO, Elastic Net and some adaptations) performed similarly, probably at a much lower computing cost. The TABLUP, which combines BayesB and GBLUP, generally did well. The simplest methods, GBLUP or Ridge Regression, and even worst, the fixed linear model, were much less efficient.

## Background

In 1990, Lande and Thompson [1] defined a two steps marker assisted selection procedure. Firstly, apparent effects of markers were estimated in a reference population. Secondly, during *n *generations, breeding values of candidates to selection were calculated from these estimated effects giving a so called Molecular Scores. These ideas, which founded the genomic selection, were more recently made operational by SNP chips which provide tens of thousands genotypes per individual. The seminal paper of Meuwissen *et al*. [2] presented a few statistical approaches of these Genomic Estimated Breeding Values (GEBV). A large literature followed, describing and comparing various methods.

These methods could be classified according to the assumption made concerning the variance of chromosome segments effects. The simplest assumption, assumed in BLUP methodology [2] or Ridge Regression [3], is that the variance of these effects is equal for all chromosome segments. However, this hypothesis is not consistent with classical genetic prior, and observations, that only a few chromosome segments contain QTL, with various extent of their effects, while most chromosome segments do not contain QTL.

Variable selection procedures were proposed to better fit this biological knowledge. In [2], a stepwise procedure, including a QTL detection step through single segments regression analyses, was envisaged in the least square framework. The efficiency for genomic evaluation of more advanced penalized regression approaches were evaluated, like sparse PLS [4], LASSO [5] or Elastic Net [6], which all allow the vast majority of loci to have null regression coefficients.

On the other hand, Bayesian methods were proposed to take into account the between chromosome segments variances heterogeneity. In BayesA [2], each chromosome segment is given its own variance, all segments contributing to the variability. This last hypothesis is made free with other Bayesian techniques which assume that only a fraction π of the segments carry QTL: BayesB keeps the between segments variance heterogeneity, while BayesC considers a single variance for the active segments. In BayesCπ, the proportion π is estimated from the data [7].

The QTLMAS XV^th ^dataset consisted of the pedigree, marker genotypes and quantitative trait performances of animals with a sib family structure [8]. Pedigree and genotypes concerned 3,000 progenies among those 2,000 were phenotyped. The trait was regulated by 8 QTLs which displayed additive, imprinting or epistatic effects. The 1,000 unphenotyped progenies were considered as candidates to selection. Participants of the XV^th ^QTLMAS workshop were invited to predict GEBV of these 1,000 individuals and to send to the organizers the description of their methods and results before the meeting. This paper aims at comparing the GEBV estimations obtained by participants to the workshop. Comparing the results obtained by the different groups should provide insight into determining which method is best fitted to analyze this kind of data set.

## Methods

### Simulated data

The simulated data set was described by Elsen *et al*. [8]. Briefly, the population comprised 3,000 individuals born from 20 sires and 200 dams *, i.e*. 10 dams per sire. Within each family, 10 progenies were assigned phenotypes and marker genotypes and 5 were assigned only marker genotypes. A total of 10,000 SNPs carried by 5 chromosomes of 1 Morgan each were simulated. Eight QTLs were simulated: one quadri-allelic additive QTL with a large effect on Chr1, two linked QTLs in phase on Chr2, two linked QTLs in repulsion on Chr3, one imprinted QTL on Chr4 and two interacting QTLs on Chr5. Random noise was added giving an heritability coefficient of 0.30. The marker density, linkage disequilibrium (LD) and minor allele frequency (MAF) were similar to real life parameters.

### Computation of the true genotypic and breeding values

"True" genetic values of the candidates to selection were calculated from simulated QTL genotypes information. Two values were calculated for each candidate. Firstly, a True Genotypic Value (TGV) defined as the sum of the 5 chromosomal genotypic values corresponding to the candidate genotypes at each of these chromosomes. The TGV of candidate *i*, *TGV_i _*depends on its QTL genotypes $\left(g_{i}^{1},g_{i}^{2}...g_{i}^{5}\right)$ and on the QTL effects (*a_j_*) given in the Table 1 of Elsen *et al*. [8]:

**Table 1:** Method*s *used by the participants to the XV^th ^QTLMAS workshop

First author	Label	Method	Description
Shariati	BayesS_1	2 steps (all SNP)	First step: a GBLUP giving estimation of SNP effects. Groups of size 150, 75 (SPNa) or 50 (SNPb) are made assembling SNP of similar effect. Second step: BayesA with all or a limited (1500 or 450) number of SNP and a unique SNP effect variance per group.
		
	BayesS_2	2 steps (1500 SNP)	
		
	BayesS_3	2 steps-Bayes (450 SNPa)	
		
	BayesS_4	2 steps-Bayes (450 SNPb)	

Ogutu	RR	Ridge regression	

	GBLUP_O	GBLUP	Qualified Ridge Regression BLUP by the authors

	LASSO_O	LASSO	

	LASSO_ad	Adaptative LASSO	Following Zou [21], data-driven weights are added to the penalty to force LASSO to be consistent

	EN	Elastic net	

	EN_ad	Adaptative EN	Mixture of adaptative lasso and EN

Wang	BayesA_W	BayesA	

	BayesB_W	BayesB	

	BayesCπ_W	BayesCπ	

	TABLUP	TABLUP	In the genomic matrix, loci IBD probability estimations are weighted by their effect variance estimated from BayesB [11]

	GBLUP_W	GBLUP	

Mucha	AM	Animal model	All models are estimating haplotypes effects. Haplotypes are obtained using the PHASE software [18]. RM1 and RM2 differ by the estimation of the haplotype effect variance
	
	FM	Fixed effect	
	
	RM1	Random model 1	
	
	RM2	Random model 2	

Zeng	GBLUPa_Z	GBLUP1	Additive effect only

	GBLUPd_Z	GBLUP2	Additive and dominance effect

	BayesB _W	BayesB	

	BayesCπ_W	BayesCπ	

Usai	LASSO_Uc	LASSO-LARS classic	The penalty is describes as ∑|*β_j_*|≤*t*. In the LASSO-LARS classic, the *t *parameter is the average number of active SNP in 1000 simulations. In strategy 1, the number which occurred more than 5% of the times and in strategy 2, which minimized a selection criteria
	
	LASSO_Uc1	LASSO-LARS strategy 1	
	
	LASSO_Uc2	LASSO-LARS strategy 2	

Schurink	BayesZ	BayesZ	Similar to BayesCπ, with a Bernoulli prior for π

$$TGV_{i}=\overset{5}{\underset{j=1}{\sum }}a_{j}\left(g_{i}^{j}\right)$$

Secondly, the expectation of the genotypic value of candidate's progenies was calculated, according to the same principle, *i.e*. as a sum of chromosomal genotypic values. It depends on the QTL genotypes of the candidate, on the QTL effects and on the frequencies of QTL genotypes in the population, *i.e*. the QTL genotypes probabilities of the mate of the candidate. This breeding value was noted *TBV_i_*:

$$TBV_{i}=\overset{5}{\underset{j=1}{\sum }}\overset{n}{\underset{g^{j}=1\;}{\sum }}prob\left(g^{j}/g_{i}^{j}\right)*a_{j}\left(g^{j}\right)$$

where the *g^j ^*are the *n *QTL possible genotypes on the chromosome *j* (*n*=(10,9,9,4,9) for the chromosomes 1 to 5 respectively), $prob\left(g^{j}/g_{i}^{j}\right)$ is the probability of the genotype *g^j ^*for the progeny of the candidate *i *given the candidate's genotype and *a_j _*(*g^j^*) is the genotypic value associated to that QTL genotype (Table 1 in [8]). Concerning the QTL6 on chromosome 4, which was imprinted, candidates were considered as those which give the allele expressed by their progeny.

The participants were sent the TGV and TBV only after the meeting.

## Methods used by the participants

The participants estimated Genomic Estimated Breeding Values, noted GEBV in the following, and sent them, with a short description of the methods used, to organizers before the meeting. A total of 27 methods were studied by the participants (table 1). Most of them belong to the three categories which were presented in the introduction: (i) (G)BLUP methods - including Ridge regression [9], GBLUP describing dominance effect [10] and TABLUP [11] where the genomic matrix includes information about the SNP effect variance (here estimated using BayesB) [12] (ii) Selection variable procedures - LASSO and Elastic Net, including adaptative versions which aim at forcing the LASSO to be consistent, *i.e*. to correctly estimate the subset of zero coefficients with a probability tending to 1 [9,13] and (iii) Bayesian approaches [12,10] - including the BayesZ [14,15] and a new two-steps Bayes procedure intermediate between the BayesA or B (one variance for each SNP) and the BayesC (a single variance for the active SNP), with a grouping of SNP based on their effect estimated with a GBLUP [16]. This method will be given the "BayesS" acronym in the following. Mucha et al. [17] used simple linear models (fixed or random) with the idea of estimating haplotype rather than SNP effects, the haplotypes being inferred with the PHASE software [18].

### Comparison criteria

Results (GEBV as given by the participants) were compared based on 4 criteria. For each criteria, the two True Values (TGV and TBV) were considered. Accuracy of GEBV was calculated as the Pearson's correlation between the TV and the GEBV. Ability to identify the best individuals was assessed from the Spearman's rank correlation between the TV and the GEBV in the top 10% of TV. Bias was assessed from the linear regression coefficient (named also the regression slope) of the TV on the GEBV. Finally, mean squared error of prediction was calculated on GEBV and TV centered on zero.

## Results

They are presented in table 2 (TGV) and 3 (TBV). The ranking is nearly the same for those two values.

**Table 2:** Comparison of True Genomic Values estimations

First author	Label	r	rank	bias	MSE
Shariati	BayesS_1	0.86	0.53	0.89	7.51
	
	BayesS_2	0.86	0.53	0.89	7.52
	
	BayesS_3	0.86	0.53	0.88	7.85
	
	BayesS_4	0.85	0.55	0.87	8.00

Ogutu	RR	0.85	0.54	1.19	8.44

	GBLUP_O	0.90	0.52	1.11	5.55

	LASSO_O	0.92	0.63	1.09	4.67

	LASSO_ad	0.92	0.62	1.02	4.30

	EN	0.92	0.62	1.23	4.96

	EN_ad	0.90	0.40	0.97	5.48

Wang	BayesA_W	0.92	0.65	1.06	4.15

	BayesB_W	0.93	0.70	1.05	3.66

	BayesCπ_W	0.93	0.70	1.06	3.63

	TABLUP	0.91	0.68	0.97	4.59

	GBLUP_W	0.78	0.37	1.20	11.71

Mucha	AM	0.61	0.36	1.06	17.57

	FM	0.49	0.32	0.35	43.17

	RM1	0.70	0.38	1.77	16.65

	RM2	0.71	0.38	1.69	16.30

Zeng	GBLUPa_Z	0.82	0.53	1.04	8.94

	GBLUPd_Z	0.81	0.52	1.04	9.46

	BayesB _W	0.93	0.71	1.05	3.63

	BayesCπ_W	0.94	0.72	1.07	3.41

Usai	LASSO_Uc	0.92	0.62	1.25	5.04

	LASSO_Uc1	0.90	0.64	1.02	5.30

	LASSO_Uc2	0.92	0.63	1.09	4.66

Schurink	BayesZ	0.90	0.60	1.06	5.20

r=Pearson correlation between TGV and GEBV, rank=rank correlation of the best 10% TGV, bias = regression coefficient between TGV and GEBV, MSEP= mean squared error of prediction of TGV by GEBV.

**Table 3:** Comparison of True Breeding Values estimations

First author	Label	r	rank	bias	MSE
	
Shariati	BayesS_1	0.84	0.48	0.33	12.92
	
	BayesS_2	0.84	0.47	0.33	12.94
	
	BayesS_3	0.83	0.49	0.33	13.37
	
	BayesS_4	0.82	0.49	0.32	13.62
Ogutu	RR	0.83	0.52	0.45	5.55

	GBLUP_O	0.81	0.51	0.39	8.23

	LASSO_O	0.87	0.55	0.43	6.44

	LASSO_ad	0.88	0.60	0.37	9.94

	EN	0.87	0.52	0.44	5.86

	EN_ad	0.81	0.48	0.33	12.01

Wang	BayesA_W	0.86	0.61	0.38	9.07

	BayesB_W	0.89	0.66	0.38	9.12

	BayesCπ_W	0.88	0.65	0.39	9.00

	TABLUP	0.88	0.64	0.36	10.88

	GBLUP_W	0.77	0.48	0.46	4.98

Mucha	AM	0.59	0.37	0.40	5.93

	FM	0.47	0.44	0.13	43.01

	RM1	0.70	0.34	0.68	2.54

	RM2	0.70	0.34	0.65	2.68

Zeng	GBLUPa_Z	0.82	0.50	0.40	7.61

	GBLUPd_Z	0.81	0.49	0.40	7.59

	BayesB _W	0.89	0.66	0.38	9.22

	BayesCπ_W	0.89	0.66	0.39	8.84

Usai	LASSO_Uc	0.86	0.53	0.45	5.66

	LASSO_Uc1	0.86	0.62	0.37	9.54

	LASSO_Uc2	0.87	0.55	0.43	6.48

Schurink	BayesZ	0.87	0.63	0.39	5.20

(r=Pearson correlation between TBV and GEBV, rank=rank correlation of the best 10% TBV, bias = regression coefficient between TBV and GEBV, MSE= mean squared error of prediction of TBV by GEBV)

### Accuracy

The Pearson correlation between GEBV and the TV were consistent within type of technique used. The range was large, from 0.49 (GEBV- TGV correlation, 0.47 for GEBV-TBV) for the Mucha et al. [15] fixed effect model to 0.94 (GEBV- TGV correlation, 0.89 for GEBV-TBV) for the Zeng et al. [10] BayesCπ. The highest correlations were obtained with the Bayesian approaches, with a very good performance of BayesCπ [10,12] which overperformed BayesZ, a similar approach based on an alternative prior. The very limited number of QTL simulated in the dataset, a situation far from the BayesZ prior, is a possible explanation for this difference. The same argument could explain the lower performance of the BayesS [16], where SNP effects are assembled in groups of similar effects. The TABLUP, which mixes BayesB estimation and GBLUP, was intermediate between the "classical" and the new Bayesian approaches of Shariati et al. [16].

The variable selection procedures can work nearly as well as the BayesB or C, in particular the LASSO and Elastic net [9,13]. However the adaptative Elastic Net did not give the expected improvement.

The GBLUP performances were more variable with a very low correlation given by the Mucha et al. [17] version based on haplotypes, and higher values for the Zeng et al. [10] and Ogutu et al. [9] proposals. Finally, the fixed effect linear model was far below all other methods.

Even if all tendencies were observed for both groups of correlations, the correlations between GEBV and the TBV were always lower than the correlations between GEBV and TGV. These last correlations were always lower than the former.

### Rank correlation

As compared to the Pearson's correlation, this criteria, which illustrates how methods can capture the best individuals, shows a similar range (0.32 to 0.72, *i.e*. 0.4 points of correlation between extreme situations), *i.e*. the fixed model and the BayesCπ. Globally the classification between groups of methods is the same: Bayes methods outperformed the selection variable approaches, the GBLUP family arrived last in the classification. The only exception was the TABLUP which was positioned between the two first groups. However, within some groups, differences were exacerbated. This was particularly true for the Bayes group, where the ranking BayesCπ > BayesB > BayesA > BayesZ > BayesS was preserved, and even more for the Ogutu et al. [9] selection variable, with a very low correlation observed for the adaptative Elastic Net. Notably the random model proposed by Mucha et al. [17] fell in the worst positions with this criterion.

The rank correlation between GEBV and TBV is generally lower than the GEBV-TGV one, with some exceptions (GBLUP [12], animal and fixed models [17], adaptative EN [9]).

### Regression coefficient (or regression slope)

Unbiased estimators are supposed to have a regression coefficient of 1. Most of the regression coefficients observed were in the range 0.85-1.25. The ranking of the Bayesian techniques were consistently correct, while the coefficient were more variable for the other approaches. Three of the methods proposed by Mucha et al. [15] clearly gave biased estimations (the fixed and both random models).

### Mean squared error of prediction (MSEP)

The results are still very consistent with the other criteria. The Bayesian techniques (excluding BayesS) and the selection variable techniques (LASSO or EN) gave the more precise estimations of the TGV. TABLUP was in the same range. The GBLUP and BayesS performed not as well and Mucha et al. haplotypes models [17] did very badly.

The MSEP of the TBV were quite different and were above or under the TGV MSEP depending on the method. The more precise estimation was given by the Mucha et al. [17] random model. LASSO, EN and GBLUP were satisfying, while the Bayesian approaches (in particular the BayesS) provided high Mean Squared Error of Prediction.

## Conclusions

The very general tendency is a better ranking of the Bayesian methods, in the alphabetic order (A<B<C<Cπ) whatever the criteria and the True Value considered (with the notable exception of the MSEP of the TBV). The Selection variable procedures (LASSO, Elastic Net and some adaptations) performed similarly, probably at a much lower computing cost. The TABLUP, which combines BayesB and GBLUP, generally did well. The simplest methods, GBLUP or Ridge Regression, and even worst, the fixed linear model, were much less efficient. The approach followed by Mucha et al. [17] to incorporate haplotype information was not efficient.

These observations are consistent with the results presented in the previous analyses of QTLMAS data [19-21], even if the genetic architecture simulated was restricted to a quite limited (8) number of QTL. It may be that this oligogenic situation did not work in favor of methods probably more suited to highly polygenic cases, such as BayesS [16] or BayesZ [15].

## Competing interests

The authors declare that they have no competing interests.

## List of abbreviations used

SNP: Single Nucleotide Polymorphism; QTL: Quantitative Trait Locus; MAF: Minor Allele Frequency; LD: Linkage Disequilibrium; GEBV: Genomic Estimated Breeding Value; TBV: True Breeding Value; TGV: True Genomic Value; LASSO: Least Absolute Shrinkage and Selection Operators; EN: Elastic Net; MSEP: Mean Squared Error of Prediction; GBLUP: Genomic Best Linear Unbiased Prediction.

## Authors' contributions

OD and OF collected and processed the data files. PLR analyzed the data. PLR and JME wrote the manuscript. All authors contributed to the ideas and methods, and read and approved the manuscript.
